# Psychometric Properties of the 12-Item World Health Organization Disability Assessment Schedule (WHODAS 2.0), Greek Version: A Cross-Sectional Study on Applicants of Welfare Benefits

**DOI:** 10.7759/cureus.48588

**Published:** 2023-11-09

**Authors:** Georgios Theotokatos, Reuben Escorpizo, Theodore J Angelopoulos, Nikolaos K Chrysagis, Jerome Bickenbach, Aikaterini Venieri, Konstantinos Karteroliotis, Eirini Grammatopoulou, Emmanouil Skordilis

**Affiliations:** 1 Physical Medicine and Rehabilitation, School of Physical Education and Sport Science, National and Kapodistrian University of Athens, Athens, GRC; 2 Rehabilitation and Movement Science, College of Nursing and Health Sciences, University of Vermont, Burlington, USA; 3 Physiotherapy, School of Health & Care Sciences, University of West Attica, Athens, GRC; 4 Physical Medicine and Rehabilitation, Swiss Paraplegic Research (Schweizer Paraplegiker Forschung), Nottwil, CHE; 5 Physical Medicine and Rehabilitation, Faculty of Health Sciences and Medicine, University of Lucerne, Lucerne, CHE; 6 Sports Excellence, 1st Orthopedics Department, School of Health Sciences, National and Kapodistrian University of Athens, Athens, GRC

**Keywords:** biopsychosocial model, social welfare, disability evaluation, icf, psychometric properties, whodas 2.0

## Abstract

Introduction: The International Classification of Functioning, Disability, and Health (ICF) provides a framework for the biopsychosocial model of disability and was developed by the World Health Organization (WHO). The World Health Organization Disability Assessment Schedule (WHODAS 2.0) is an ICF-based tool that measures health and disability at the population level or in clinical practice. The aim of the study was to examine the psychometric properties of the Greek version of the WHODAS 2.0 (12-item) administered to 10,163 adults who had applied for welfare benefits in three regions of Greece.

Methods: The WHODAS 2.0, administered by interview was the primary outcome variable. Principal axis factoring (PAF) and confirmatory factor analysis (CFA) assessed the data fit to the model (construct validity). The correlation between Barema disability percentage (assessed by a three-member medical committee) and WHODAS 2.0 score and the correlation between WHODAS 2.0 score and the number of comorbidities were also examined (concurrent validity). Cronbach’s alpha was used to assess the internal consistency of the questionnaire. Floor and ceiling effects were also examined.

Results: Internal consistency was acceptable (Cronbach’s alpha=0.918). A significant association was found between Barema disability percentage and the WHODAS 2.0 score. Factor analysis showed a clear two-factor solution (PAF and CFA), while no floor or ceiling effects were evident.

Conclusion: The Greek version of the 12-item WHODAS 2.0 was found to be reliable and valid in a wide sample of applicants for welfare benefits.

## Introduction

In 2001, the World Health Organization (WHO) developed the International Classification of Functioning, Disability, and Health (ICF), a biopsychosocial model of disability describing individual functional levels, regardless of health conditions [1]. The ICF contextual factors (environmental and personal) are part of the model; the interaction between these factors is dynamic and bidirectional, and the environmental and personal factors influence the health condition and vice versa. The development of the ICF as a reference framework was a stepping stone for researchers, policymakers, and rehabilitation experts and it was expected to enhance multidisciplinary collaboration [2].

The ICF framework was used by the WHO as the basis for the Disability Assessment Schedule (WHODAS 2.0), a practical assessment tool of health and disability in general and for specific clinical populations [3]. There are various versions of the tool: long (36-items), short (12-items), self-administered, interview-administered, or proxy versions [3]. The WHODAS 2.0 assesses the functioning of an individual in six different domains of daily life by scoring the difficulty of the person in (1) understanding and communicating (cognitive function), (2) getting around (mobility), (3) self-care, (4) getting along with people, (5) life activities, and (6) participation in society. The overall score represents a summary of the whole person's experience of disability, while the underlying domain scores in the long-form version can be used to assess the limitations of the respective domains of daily life [3]. The WHODAS 2.0, as highlighted by Ustün et al., (2010), may be used irrespective of the underlying health conditions and can be administered by healthcare professionals in various settings in order to enhance clinical practice, research, education, and policy development [3].

The WHODAS 2.0 has been translated into 47 languages and used across several cultures and age groups, as well as for different types of diseases and health conditions (e.g., psychiatric and mental disorders, Alzheimer’s disease, geriatric populations, patients with peripheral inflammatory arthritis, trauma patients, or in various disability and rehabilitation settings) [4-6]. A systematic review conducted in 2017 by Federici et al. identified 810 relevant studies, from 94 countries, that were published between 1999 and 2015, where the WHODAS 2.0 was used for assessing functioning and disability in different settings and individual health conditions [4], in surveys [7,8], registries [9], for monitoring patients’ outcomes in rehabilitation settings and clinical practice [10-12]. 

So far, several international studies have established the psychometric properties of the short version of the WHODAS 2.0 to different populations (e.g., Israel, USA, Nepal, China, Poland, Singapore) [13-17]. Similarly, three separate studies were conducted in Greece to assess the psychometric properties of the WHODAS 2.0. Xenouli et al. (2016) examined 101 individuals with chronic diseases receiving disability allowance and 109 individuals without disabilities [18]. The researchers reported sufficient Cronbach’s alpha, construct, and concurrent validity evidence. However, the exploratory factor analytic results revealed a five-factor solution for the group without disability and a four-factor solution for the group of individuals receiving disability allowance. Papadopoulou et al. (2020) explored the psychometric properties of the Greek WHODAS 2.0 version in 136 adults with motor disabilities [19]. The researchers reported high reliability, high to low concurrent validity evidence, and a three-factor solution through exploratory factor analysis. Finally, Koumpouros et al. (2018) provided cross-cultural validity evidence for the Greek WHODAS 2.0 long-form version. The researchers examined 200 participants from the general population (aged over 61 years old) in a Greek Open Care Center for the elderly and stated that the validity and reliability evidence were sufficient [20].

The studies conducted in Greece so far have mainly used a limited number of participants and health conditions (101 to 200 individuals with either chronic diseases, motor disabilities, or from the general population). However, the sample-specific validity evidence theory suggests that these findings may not be generalizable, and therefore more research is required in Greece to assess the psychometric properties of the WHODAS 2.0 Greek version [21, 22]. According to Sherrill and O’Connor (1999), different samples do not necessarily respond the same way to different instruments and protocols [21], while Yun and Ulrich (2002) stated that validity and reliability may not be generalized and should always be examined [22]. Cook and Beckman (2006) suggested that validity and reliability assessment of clinical tools such as questionnaires, orthopedic tests, and observational studies permit a sound interpretation of the results [23]. Hence, the aim of the present study was to examine the psychometric properties of the WHODAS 2.0 short-form, interview-administered Greek version on a wide sample of individuals and various health conditions who had applied for welfare benefits in Greece. Specifically, we examined the internal consistency, the convergent and construct validity, and floor and ceiling effects. Based on previous ambiguous findings (2-5 factors), the ICF theory, and the respective environmental and personal factors, a two-factor solution was anticipated to represent the WHODAS 2.0 data.

## Materials and methods

Participants and procedures

Individuals with disabilities from three main regions in Greece (Athens, Thessaloniki, and Patra) who had submitted an application for welfare benefits to the Ministry of Labor and Social Solidarity (MoLSS) were recruited. Participants 18 years of age or older who were able to comprehend and speak Greek fluently were eligible to participate. All the non-adult participants were excluded from the analyses. The present study was a continuation of a pilot project, initiated by the MoLSS, the World Bank (WB), and the European Commission (EC) Directorate-General (DG) for Structural Reform (who funded, to an extent, and supervised the program). The results of the WB’s report can be found elsewhere [24]. The permission for assessing the participants had been provided by the Greek state, and specifically from the MoLSS. All procedures performed in this study involving the human participants were in accordance with the ethical standards of the 1964 Helsinki Declaration and its later amendments or comparable ethical standards. 

In Greece, the certification of disability percentage is decided upon a three-member medical committee assessment. The specializations of the doctors participating in the committees are related to the primary condition of the applicants and are responsible for assessing disability in the Greek welfare system. The committees assess the medical history, recent surgeries or hospitalizations, pharmaceutical treatments, rehabilitations and/or interventions, medical tests or screenings of the patients according to the ICD-10. In the present study, in addition to the three-member medical committee, a fourth member (another medical doctor), administered the interview short-form version of WHODAS 2.0 independently, before or after the medical assessment. Previous evidence suggested that the WHODAS scores tend to be higher for participants seeking or receiving compensation than those who are not [25]. Hence, the participants in the present study were assured that the WHODAS 2.0 responses would not affect the decision of the three-member medical committee on their disability assessment and welfare benefit eligibility, in order to prevent self-reporting bias.

The 12-item version of WHODAS 2.0 was administered by interview. The extended version of the WHODAS consists of 36 items and the average interview is 20 minutes. The average interview time for the interviewer-administered 12-item version is five minutes. Due to administrative limitations, there was no capacity to administer the 36-item version. As described in the procedure section, the applicants were also examined by the three-member medical committee and it would be a burden for them to have a 20-minute interview before or after the medical examination. In total, 36 medical doctors served as interviewers in three separate centers for assessing individual applications for disability-related welfare benefits. All medical doctors went through workshops from a panel of WHODAS 2.0 experts (members of the WB) on how to administer the measure and follow the guidelines.

Instruments

The Greek version of the WHODAS 2.0 short-form version (12 items) [20], administered by interview, constituted the primary outcome variable. Based on the guidelines of the WHODAS, items determine how much difficulty the participants had in various daily activities. A five-point Likert scale is used for rating the difficulty and the possible responses are as follows: 1=none, 2=mild, 3=moderate, 4=severe, 5=extreme or cannot do. Hence, higher WHODAS scores correspond to more limitations and restrictions. Hence, no difficulties correspond to no limitation (and a minimum WHODAS score of 0%) and extreme difficulties correspond to many restrictions (and a maximum WHODAS score of 100%). 

The WHODAS respondents should reply taking into consideration the tool’s points of reference. More specifically, the degree of difficulty they experience, where difficulty is considered as increased effort, discomfort, pain, slowness, and changes in the way they do given activities. In addition, they should only reply about how much difficulty they face due to their health conditions and not any other reason. Recall bias led the developers to select the period of one month as the timeframe for WHODAS 2.0. Recall abilities are most accurate for the period of the past 30 days and averaging the good and bad days. Another frame of reference is “as the respondent usually does the activity”. For example, if assistive equipment or personal assistants are available, they should reply keeping this help in mind. This study was implemented with interviewers not evaluating the added value of personal or technical assistance. The interviewers asked the questions once, advising the participants to take into consideration the available help. Finally, the items are rated as not applicable if the person did not actually do the activity during the period of the last 30 days. When linguistic barriers or cognitive restrictions that hindered the interview process appeared, the proxy version of the questionnaire was used (completed by relatives, carers, etc.).

The decision of the medical committee on the disability percentage (ranges 0-100%, where 100% is considered as a totally disabled person) is based on the Unified Table for Determination of Disability Rate (UTDDR) that serves as a point of reference for the International Classification of Diseases-10th edition (ICD-10) and the respective disability percentages for each health condition (Barema Disability Percentage). Historically, the Barema scale has been used to attribute disability percentages to war survivors/veterans or work-related accidents (e.g., 5% for the amputation of a finger, 50% for the amputation of the lower extremity, etc.) [26].

Statistical analyses

The Statistical Package for the Social Sciences (SPSS v. 28, IBM Corp., Armonk, NY) and the AMOS software (Amos Development Corp., Meadville, PA) were used [27]. All the descriptive statistics were examined for normality (histogram, skewness, kurtosis). The responses were examined for floor and ceiling effects (if more than 15% of the patients scored the minimum and maximum possible score respectively [28]). The internal consistency of the items was examined with Cronbach’s alpha coefficient. The convergent validity of the questionnaire was examined with Pearson's r and the correlations between the WHODAS 2.0 score and the (a) medical assessment (Barema scale) and (b) the number of morbidities reported by the applicants. The correlations <0.20 were considered low, 0.20-0.30 low-moderate, 0.31-0.50 moderate, and >0.50 moderate-to-high [29].

Subsequently, the WHODAS 2.0 structure was examined through (a) principle axis factoring (PAF) and (b) confirmatory factor analysis (CFA). The decision to use both PAF and CFA was based on the split sample approach, as an important step for building confidence in the validity of the findings [30-32]. The whole process requires two samples from the same population, randomly split: the first sample to establish the baseline model, and the second sample for cross-validation, using structural equation modeling techniques such as CFA. The split method has been reported by several researchers in the past and was considered appropriate for the purposes of the present study (e.g.: 20,22). A two-factor model was hypothesized then, with oblique rotation (correlated factors), according to the ICF theory (factors: environmental and personal). Bartlett’s test of sphericity and the Kaiser-Meyer-Olkin (KMO) criterion examined the data adequacy for PAF. According to Grieder and Steiner (2022), the PAF is a fitting approach to exploratory factor analysis, using the variance/covariance of indicators to extract a pre-specified number of factors explaining maximum variability [33]. According to Fabrigar et al. [30], the PAF is one of the most popular used exploratory factor analytic methods, known to identify hypothesized factors with wide samples. In the present study, the 0.40 criterion for factor loading (FL) was used, along with eigen values above 1.00 and the respective scree plot. Only items with FL > 0.40 in a hypothesized factor were retained [34].

Further, the factorial structure that best fits the data was examined with confirmatory factor analysis (CFA) [27]. The items were allowed to load on appropriate factors in the CFA, according to the previous PAF results. Absolute and incremental fit indices estimated the sufficiency of the model. The absolute fit indexes evaluated how well the prior two-factor WHODAS 2.0 model reproduced the data [35]. The chi-square (χ2) statistic and the root mean square error of approximation (RMSEA) served as the absolute fit indexes. In turn, the non-normed fit index (NNFI), incremental fit index (IFI), comparative fit index (CFI), Tucker-Lewis index (TLI), and goodness of fit index (GFI) represented the incremental fit indexes [36-38]. The incremental fit indexes were used for comparing the fit of the WHODAS 2.0 model with a null model [39]. The covariance between errors was set to zero and the maximum likelihood (ML) procedure was used to estimate the model [39]. The statistical significance of this study was set to p < 0.05.

## Results

Demographics/descriptive statistics/floor and ceiling effects

A total of 14,113 cases were initially considered. Accordingly, the non-adult cases were removed (N=1,834). During the process of the medical disability evaluation, certain applicants appealed for a second assessment. In these cases, the WHODAS 2.0 was only administered once and only the medical result was subject to change. There were 398 duplicate cases; all were excluded and removed from subsequent analyses. The research team decided to consider only complete cases [40] and excluded all the cases with missing data or non-applicable items (N=1,718). The final sample therefore constituted 10,163 individuals. The respective demographic characteristics and the descriptive statistics (means and standard deviations) for WHODAS 2.0 and the Barema disability percentage are in Table 1. No floor or ceiling effects were observed. The lowest score of WHODAS 2.0 was found in 0.74% of the participants (N=75) and the highest score was found in 0.99% of the participants (N=101).

**Table 1:** Demographics and clinical characteristics of the study’s sample.

Variable				N (%)
Gender	Male		4,406 (43.35)	
	Female		5,757 (56.65)	
Education (Years of studies)	0-6 years		3,105 (30.55)	
	7-12 years		4,403 (43.33)	
	>12 years		2,655 (26.12)	
Living Condition	Independent in the community		8,191 (80.6)	
	Assisted living		1,939 (19.08)	
	Hospitalized		33 (0.32)	
Marital Status	Never married		3,747 (36.87)	
	Currently Married		4,299 (42.3)	
	Separated		386 (3.8)	
	Divorced		1,094 (10.77)	
	Widowed		543 (5.34)	
	Cohabitating		94 (0.92)	
Work Status	Paid work		578 (5.69)	
	Self-employed		233 (2.29)	
	Non-paid work		30 (0.3)	
	Student		435 (4.28)	
	Keeping house		2,586 (25.45)	
	Retired		497 (4.89)	
	Unemployed (Health-reasons)		4,259 (41.9)	
	Unemployed (Other reasons)		1,423 (14)	
	Other		122 (1.20)	
Number of Morbidities		1	6,476 (63.73)	
		2	2,042 (20.09)	
		3	921 (9.06)	
		4	426 (4.19)	
		5	232 (2.28)	
		6	44 (0.43)	
		7	19 (0.19)	
		8	3 (0.03)	
Version		Non-proxy	8,726 (85.86)	
		Proxy	1,437 (14.14)	
Age (years)		Mean (SD)	35.13 (21.20)	
WHODAS 2.0, 12-item score		Mean (SD)	50.26 (24.26)	
Barema Disability Percentage		Mean (SD)	65.10 (16.20)	

Reliability measures

The internal consistency of the WHODAS 2.0 had an overall Cronbach’s alpha coefficient of 0.918 (Table 2).

**Table 2:** Internal consistency of the 12-item WHODAS 2.0 questionnaire.

Item	Domain	Cronbach's Alpha if Item Deleted
S1. Standing for long periods such as 30 min?	Mobility	0.913
S2. Taking care of your household responsibilities?	Life activities	0.907
S3. Learning a new task, for example, learning how to get to a new place?	Cognition	0.91
S4. How much of a problem did you have joining in community activities (for example, festivities, religious or other activities) in the same way as anyone else can?	Participation	0.909
S5. How much have you been emotionally affected by your health problems?	Participation	0.916
S6. Concentrating on doing something for ten minutes?	Cognition	0.91
S7. Walking a long distance such as a kilometer [or equivalent]?	Mobility	0.913
S8. Washing your whole body?	Self-care	0.909
S9. Getting dressed?	Self-care	0.91
S10. Dealing with people you do not know?	Getting along	0.912
S11. Maintaining a friendship?	Getting along	0.914
S12. Your day-to-day work/school?	Life activities	0.909
Overall		0.918

Convergent validity

The Pearson’s correlation between the WHODAS 2.0 disability percentage and the Barema disability percentage was low-moderate (Pearson’s r = 0.243, p<0.0001). Additionally, the correlation between the WHODAS 2.0 disability score and the number of morbidities was also low-moderate (Pearson’s r = 0.213, p < 0.001).

Construct validity/PAF and CFA

The split sample approach separated randomly the total sample of 10,163 participants into two parts. The first part (N=5,081) was used for the PAF and the second part (N=5,082) for CFA. Subsequently, the PAF with an oblique rotation provided a clear two-factor solution according to the scree plot, with eigen values greater than 1, explaining 58.376% of the total variance. All factor loadings had values above 0.40. The seven items (items S3, S4, S5, S6, S10, S11, S12) of the first factor, accounted for 33.502% of the total variance with an eigen value of 4.020. The second factor, with five items (items S1, S2, S7, S8, S9), accounted for 24.874% of the variance and the respective eigen value was 2.985. The results of the PAF, with the respective factor loadings, eigen values, and percentages of explained variability may be found in Table 3.

**Table 3:** Results from the PAF Eigen values: Factor 1=4.020, Factor 2=2.985 % of explained variability: Factor 1=33.502, Factor 2=24.874 Cronbach’s a: Factor 1=.887, Factor 2=.892

Item	Factor 1	Factor 2
S1. Standing for long periods such as 30 min?	-	0.819
S2. Taking care of your household responsibilities?	0.424	0.458
S3. Learning a new task, for example, learning how to get to a new place?	0.735	-
S4. How much of a problem did you have joining in community activities (for example, festivities, religious or other activities) in the same way as anyone else can?	0.574	-
S5. How much have you been emotionally affected by your health problems?	0.419	-
S6. Concentrating on doing something for ten minutes?	0.775	-
S7. Walking a long distance such as a kilometer [or equivalent]?	-	0.834
S8. Washing your whole body?	-	0.696
S9. Getting dressed?		0.701
S10. Dealing with people you do not know?	0.831	-
S11. Maintaining a friendship?	0.825	-
S12. Your day-to-day work/school?	0.547	-

**Figure 1:**
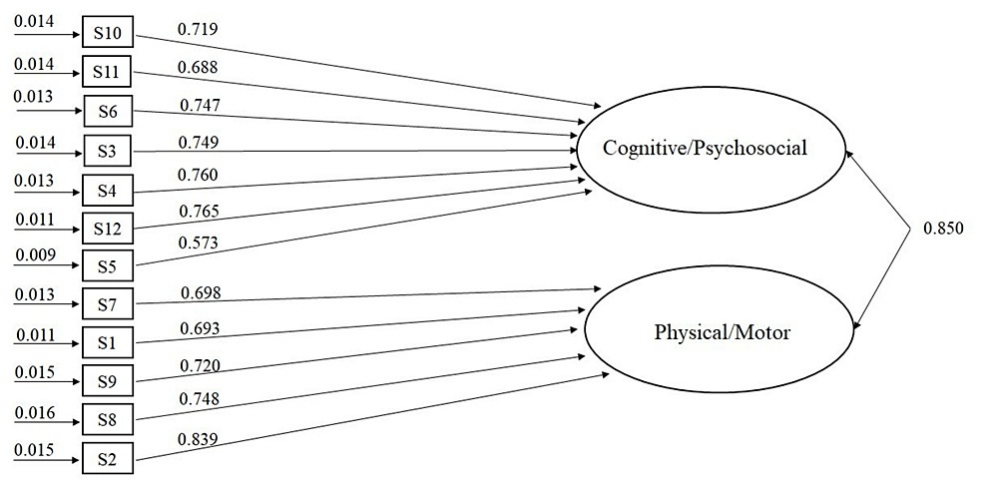
The error variances, the standardized regression weights and the intercorrelation between the two factors (cognitive/psychosocial and physical/motor).

Accordingly, the criteria of univariate skewness > + 2.00, kurtosis > + 5.00 [41,42] and Mardia’s multivariate non-normality index of kurtosis [< p (p + 2)] [43] were examined and the results were at the appropriate range. The subsequent CFA revealed a proper fit of the data to the WHODAS 2.0 model, with two factors. The chi-square was significant, while the absolute fit index (RMSEA) was 0.08. In turn, the incremental fit indexes (NNFI, IFI, CFI, TLI, GFI) were above 0.90, indicating a proper fit. The CFA results can be found in Table 4. The error variances, the intercorrelation between the two factors, and the standardized regression coefficients may be found in Figure 1.

**Table 4:** Results from the CFA. Cronbach’s a: Factor 1=.886, Factor 2=.882 GFI: goodness of fit index; NNFI: non-normed fit index; IFI: incremental fit index; TLI: incremental fit index; CFI: comparative fit index; RMSEA: root mean square error of approximation

Fit Index	Value
χ^2^	3857.398
df	51
GFI	0.933
NNFI	0.948
IFI	0.949
TLI	0.934
CFI	0.949
RMSEA	0.086

## Discussion

The present study examined the psychometric properties of the 12-item Greek version of WHODAS 2.0 in a large sample of 10,163 individuals who exhibited a variety of health conditions and had applied for welfare benefits. The WHODAS 2.0 was based on the ICF’s biopsychosocial framework and may serve as a point of reference for different disability and healthcare professionals, agencies (e.g. policy or government), and stakeholders, providing a common language of understanding and criteria for work capacity and functional status [44]. The data analyses provided acceptable construct validity, convergent validity, reliability evidence, and absence of floor and ceiling effects. The present findings come in accordance with previous studies in Greece [18,19] which had a limited number of participants and health conditions. The results are also comparable to previous international studies that examined the psychometric properties of the WHODAS 2.0 to a variety of health conditions, such as major depressive episodes [45], persons with PTSD [15], Huntington's disease [14], patients with hand injuries [13], mild traumatic injury [25], colorectal cancer survivors [46], and Kushin-Beck syndrome [47].

Reliability evidence

In the present study, Cronbach’s alpha coefficient was 0.918. The high internal consistency suggested that the 12-item Greek version of WHODAS 2.0 performed well [48] in measuring disability in patients with various health conditions. Previous studies reported moderate to high internal consistency and Cronbach’s alpha ranges (from 0.79 to 0.94) [13,14,17-19,45-47], therefore supporting the present findings.

Floor or ceiling effects

No floor or ceiling effects were found in the present study. Less than 1% reported no disability at all (N=75) and approximately 1% reported total disability (N=101). Similarly, no floor or ceiling effects were found in Papadopoulou’s [19], Schiavolin’s [49], and Snell’s [25] studies either. Contrary to the present findings, Ćwirlej-Sozańska et al. (2020) reported a floor effect that may be attributed to the non-clinical sample used (adult urban residents) [16]. Gaskin et al. suggested that a high percentage of participants with no disability (i.e., zero scores) may result in heavily censored data, generating floor effects [50]. Katajapuu et al. reported floor effects within the subscales “understanding and communicating” and “getting along with people” [51]. The limited sample of Katajapuu et al. (N=85) were patients with rheumatoid arthritis (mean age 61 years old, ranging from 22 to 83) and the researchers speculated that their cognitive or social skills might have not been affected by the disease. Overall, the absence of floor or ceiling effects in the present study, from a wide sample of applicants for welfare benefits and different health conditions, may be indicative of acceptable content validity evidence [28].

Convergent validity evidence

**Figure 2:**
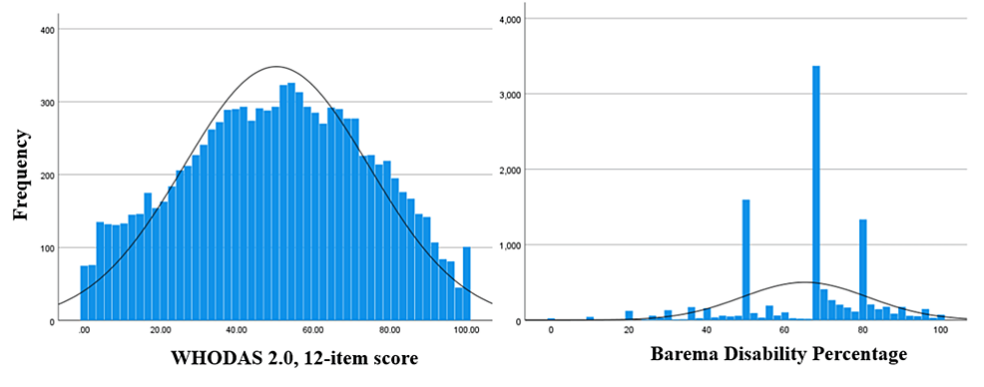
The histogram of the disability percentages based on the WHODAS 2.0 12-item score and the Barema Disability Percentage, with normal curve.

The WHODAS 2.0 responses and the medical committee’s disability percentage exhibited a significant but low-moderate association (r=0.243) with approximately 6% of common explained variability. The low-moderate association may be initially explained by the fact that the above measures assess disability from a different theoretical point of view (biomedical vs. biopsychosocial). In addition, in large sample sizes, a Pearson’s r close to zero may be significant, despite the fact that the linear association between two variables is weak and a significant correlation does not necessarily mean that a strong linear association exists [52]. In this case, the inspection of the scatter plot of the two variables when investigating the strength of a linear association is suggested [52]. The scatter plot between WHODAS 2.0 scores and Barema disability scores in the present study showed that there is no homoscedasticity. More specifically, the WHODAS 2.0 data exhibited a normal distribution of scores, while the data from the Barema Scale (medical committees’) were mainly kurtotic and certain disability percentages were repeated frequently (Figure 2).

In an attempt to interpret the non-normal distribution of the Barema scale, we noted that certain disability percentages frequently reported were 50% (N=1,579, 25.64%), 67% (N=2,863, 28.17%), and 80% (N=1,276, 12.56%). The cumulative percent of the above percentages (50%, 67%, and 80%) was 66.37% of the overall recordings by the medical committees (N=5,718). According to Greek legislation, certain percentages of disability are assigned to specific health conditions by the medical committees. The applicants with diabetes type 1 or HIV-related health conditions, for example, are by default (based on the UTDDR) assigned 50% of the disability percentage. Other health conditions (e.g., schizophrenia or lung non-operational cancer) are assigned with higher disability percentages (≥67% and ≥80% respectively) and the applicants are entitled to receive welfare benefits and certain services by the state. These figures (50%, 67%, 80%) appear at the peak in the histogram of the disability percentage of the medical committee evaluation. The description of the Greek disability assessment system is not in the scope of the present study, nevertheless, the fact that the Barema scale provides pre-determined disability percentages for specific conditions is highly debated. The Council of Europe (2002) has expressed its objections, especially when the Barema scale is used for cross-country comparisons [26].

The results of the study indicated that the 12-item Greek version of WHODAS 2.0 was significantly associated with multimorbidity. The correlation, nevertheless, was low. Similar results have been found in the past [17]. The low correlation may be attributed to the fact that the WHODAS 2.0 was designed to assess the restrictions and limitations that an individual faces in daily life, irrespective of the number of medical diagnoses [3]. In addition, the health conditions do not provide the whole picture with respect to how an illness or injury affects an individual’s daily life [53]. The biopsychosocial approach of the ICF reflects the complex notion of disability, integrating the biomedical and the social aspects of the person and taking into consideration different domains, such as education, employment, and the community in which the person is living [54].

Construct validity

Previous studies examining the factorial structure of the WHODAS 2.0 [14,16,17,45,50], with data from a variety of individuals in different settings, have attained ambiguous findings. Luciano et al., (2010) [45] for example assessed 3,615 primary care patients with depressive episodes in Spain and reported sufficient Cronbach’s alpha, convergent, and discriminant validity evidence. The researchers stated that their exploratory and confirmatory factor analytic results revealed a single-factor solution for the WHODAS 2.0 construct. Carlozzi et al. (2015) examined the responses of 477 patients with Huntington's disease in the USA [14]. The researchers reported a notable ceiling effect for almost 20% of their sample, sufficient Cronbach’s alpha coefficient, known groups and convergent validity evidence, and a six-factor solution through confirmatory factor analysis. Gaskin et al. (2017) assessed 750 individuals from several countries (China, Ghana, India, Mexico, Russia, and South Africa) and stated that their data produced a two- or three-factor solution, depending on the distribution of the samples used in the respective factor analysis [50]. In 2020, Ćwirlej-Sozańska et al. examined 584 urban residents in Poland and found high internal consistency, sufficient convergent and criterion validity evidence [16], and reported a six-factor solution through confirmatory factor analysis. Finally, Subramaniam et al. (2020) assessed 2,564 adults above 60 years in Singapore [17] and reported high internal consistency, sufficient concurrent and convergent validity evidence, and a single-factor solution through confirmatory factor analysis.

The present findings attempted to clarify the factorial structure of the 12-item WHODAS 2.0 and shed more light on the field. The ICF theory used, with two extracted factors (environmental/personal), was confirmed through principal axis factoring (PAF) and confirmatory factor analysis (CFA). The first factor (items S10, S11, S6, S3, S4, S12, S5) consisted of items from the “getting along”, “cognitive”, “participation” and “life activities” domains. The second factor (S7, S1, S9, S8, S2) consisted of items from the “mobility”, “self-care” and “life activities” domains respectively. The detailed examination of the item content led us to speculate that the first factor reflected the “cognitive/ psychosocial” and the second factor reflected the “physical/ motor” requirements of daily living. Specifically, the content examination of separate items attempted to assess more in-depth and explain theoretically the extracted factors (psychosocial/cognitive and physical/motor). 

Items from the “Getting along” domain, were classified under the first factor (psychosocial/cognitive). The wording of the respective items addressed how much difficulty they had “dealing with people they do not know” (S10), such as service personnel, shopkeepers, etc., and “maintaining a friendship” (S11). Items S3 and S6 (cognition domain of the WHODAS 2.0) were also classified under the psychosocial/cognitive factor. S3 (“Learning a new task, for example, learning how to get to a new place”?) and S6 (“Concentrating on doing something for ten minutes?”) referred to their daily ability to understand a new task and focus on something. The last two items of the first factor are S4 and S5 [“How much of a problem did you have joining in community activities (for example, festivities, religious or other activities) in the same way as anyone else can?” and “How much have you been emotionally affected by your health problems?”, respectively)]. These items (S4 and S5) probe whether the respondents had any inhibitors to joining the community’s activities and whether they had an emotional impact due to their health condition (positive or negative). Finally, the items belonging to the “life activities” domain (S2: taking care of household responsibilities; S12: day-to-day work/school) were classified into separate factors in the present study. In the WHODAS 2.0 administration guidelines, the items intend to elicit respondents’ appraisal of “any difficulty they encounter in maintaining the household and in caring for family members or other people they are close to’’, including physical, emotional, financial, or psychological needs. The responses however may differ, with respect to the respondent’s cultural differences. In Greece for example, male respondents may not have household responsibilities often, and household may be broadly defined and referring to the upkeep and maintenance of their belongings, managing finances, repairs, disciplining children, etc. Item 2, therefore, was perceived from a physical point of view, of the things they were obliged to take care of in their household. In turn, item S12 was perceived from a psychosocial/cognitive point of view, since it refers to the things that they were expected to do by a household member/supervisor/teacher, etc.

The items of the mobility domain (S1, S7) of WHODAS 2.0 were loaded under the second factor (physical/motor). These items inquire about how much difficulty they had to stand or walk. Furthermore, S8 and S9 items were also classified under the second factor and they concern the self-care of the interviewed. More specifically, S8 and S9 addressed how much difficulty they had to dress or wash their whole body, which apparently, were seen from the physical requirements point of view.

Based on the ICF’s manual, there are contextual factors that interact with the health condition, the body structures and functions, and the individual involvement in activities and community participation [1]. The contextual factors, classified as either environmental or personal, led us to hypothesize a two-factor solution in the present study. Based on the present statistical analyses and the way the items were grouped, the two extracted factors could be renamed as psychosocial/cognitive and physical/motor respectively.

Previous studies reached conflicting findings with respect to the extracted factors and the items classified accordingly [14,16-19,45,47,50]. For example [14,16,47] reported a six-factor solution from data from the short version of WHODAS 2.0. Papadopoulou et al. disputed the above argument and claimed that the short version, recruiting two items from each long-form domain is rather questionable with respect to the adequate explanation of each and every factor [19]. Papadopoulou et al. suggested that a three-factor solution may be more appropriate (motor, participation, and self-care factors). 

Gaskin et al. (2017) reported that sample selection influences the factor analytic findings and claimed that the unidimensional factor analysis is a consequence of sample selection [50]. Hence, the sample that includes people with severe disabilities will produce more factors than samples with mild or without disabilities. The above speculation was not supported in the present study, since the recruited wide sample exhibited a variety of health conditions. The difference may be attributed to the fact that Gaskin’s sample was heterogeneous with a wide number of participants with no disability, leading possibly to floor effects and ambiguous two or three-factor solutions.

Limitations/future studies

Certain limitations do not allow generalization of the results without caution. With respect to the concurrent validity evidence, the non-normal distribution in the Barema Disability Percentage might have affected the magnitude of correlation with the WHODAS 2.0 [54]. The application of other clinical/functioning tests was not possible; hence the convergent validity was only examined by testing the correlation of the WHODAS 2.0 scores to the Barema scale score and the number of different health conditions each participant had. For example, handgrip dynamometer, other questionnaires on quality of life, or validated questionnaires specific to health conditions [3,55] that have been used as gold standards for concurrent or convergent validity evidence, might have been useful.

In addition, the design of the present study did not include test-re-test reliability evidence. The cross-sectional nature of the survey was based on the capacity of the stakeholders and was not possible, on an administrative basis, to administer the questionnaire more than once. Further, the design did not test for changes in clinical practice and/or the sensitivity of change. Finally, the cultural differences between settings and/or countries may constitute another limitation to consider. The housekeeping items, for example, reflect cultural backgrounds, attitudes, and beliefs and may be considered to describe the differences in the literature examining the WHO DAS 2.0 factorial structure so far. 

Future researchers may consider examining the test-retest reliability of the Greek short-form version of the WHODAS 2.0. Testing the validity of the full WHODAS 2.0 version on a wide sample containing those with various health conditions may be useful as well. Finally, the discrepancies between the biopsychosocial (WHODAS 2.0) and the biomedical (Barema) assessments may be a reason to incorporate them both in a unified biomedical and biopsychosocial approach to functioning assessment. The above unified and holistic approach will be feasible with valid and reliable tools confirmed in future studies.

## Conclusions

The psychometric properties of the the interview-administered WHODAS 2.0 (12-items) Greek version were established in a wide sample of welfare applicants, with a variety of health conditions and levels of functioning. Following the ICF theory, the environmental and personal factors were the main determinants of functioning assessment confirming our research hypothesis. The present findings outline the importance of providing a short, quick, and suitable measure during the disability assessment process in an interview format. Further, analyzing and interpreting the results of the assessment of welfare benefits applicants with both biopsychosocial and biomedical approaches appears to be a promising endeavor from now on. Future policymakers and stakeholders may consider assessing applicants for welfare benefits with a holistic approach, combining the biopsychosocial and the medical model. In addition, the WHODAS 2.0 may be used as a basis to explore (a) in-between countries comparisons, (b) the differences between the biopsychosocial and medical approaches, (c) the long-term benefits of the holistic assessment approach for individuals and communities alike and (d) the cost-effectiveness of several treatments.
